# ESB-Based Sensor Web Integration for the Prediction of Electric Power Supply System Vulnerability

**DOI:** 10.3390/s130810623

**Published:** 2013-08-15

**Authors:** Leonid Stoimenov, Milos Bogdanovic, Sanja Bogdanovic-Dinic

**Affiliations:** Faculty of Electronic Engineering, University of Niš, A. Medvedeva 14, Niš 18000, Serbia; E-Mails: milos.bogdanovic@elfak.ni.ac.rs (M.B.); sanja.bogdanovic.dinic@elfak.ni.ac.rs (S.B.-D.)

**Keywords:** Sensor Web, electric power supply sensors, omnibus model, sensor data fusion

## Abstract

Electric power supply companies increasingly rely on enterprise IT systems to provide them with a comprehensive view of the state of the distribution network. Within a utility-wide network, enterprise IT systems collect data from various metering devices. Such data can be effectively used for the prediction of power supply network vulnerability. The purpose of this paper is to present the Enterprise Service Bus (ESB)-based Sensor Web integration solution that we have developed with the purpose of enabling prediction of power supply network vulnerability, in terms of a prediction of defect probability for a particular network element. We will give an example of its usage and demonstrate our vulnerability prediction model on data collected from two different power supply companies. The proposed solution is an extension of the GinisSense Sensor Web-based architecture for collecting, processing, analyzing, decision making and alerting based on the data received from heterogeneous data sources. In this case, GinisSense has been upgraded to be capable of operating in an ESB environment and combine Sensor Web and GIS technologies to enable prediction of electric power supply system vulnerability. Aside from electrical values, the proposed solution gathers ambient values from additional sensors installed in the existing power supply network infrastructure. GinisSense aggregates gathered data according to an adapted Omnibus data fusion model and applies decision-making logic on the aggregated data. Detected vulnerabilities are visualized to end-users through means of a specialized Web GIS application.

## 1. Introduction

Contemporary trends have caused a majority of socially responsible utility companies to aim at contributing to society through ensuring consistent quality of services. This includes electric power supply companies, which are currently facing energy crises and trying to overcome the challenges posed by the need for efficient energy utilization. For this reason, these companies are constantly seeking mechanisms that would improve their reliability and enhance the quality of the electric power supply. These mechanisms usually start with improving power supply reliability throughout improved monitoring, automation and information management. In addition, electric power supply companies have noticed that the quality of supply could be improved by shifting their business practices from employee-based knowledge to systems-based knowledge by exploiting information management and automation methods. In this business practice shift process, enterprise IT systems and applications, capable of providing an accurate power network state, have become a critical element for enhancing overall operational efficiency and system reliability.

Electric power supply companies depend on a significant number of enterprise IT systems to provide them with a comprehensive overview of the state of the distribution network, including current consumption, power network load and the status of individual network elements. At a single power supply company level, enterprise IT systems are typically interconnected with various field devices, controls and metering devices within a utility-wide network. Each IT system is dedicated to a subset of the company's operations in terms of monitoring and control. Since daily decision making and operations require a comprehensive overview of the state of the distribution network, the need for integration of information originating from different IT systems is inevitable. This kind of integration on the company level is recognized as Enterprise Information Integration (EII) and it can be based on different architectures enabling system interoperability, information management and real-time data integration as key benefits [1,2]. Recently, with the emergence of XML as a standard communication language, EII solutions are getting re-branded as products belonging to the Enterprise Service Bus (ESB) solutions group [3]. Regardless of the classification used, from the utility company's point of view, the utmost goal remains unaltered: enable daily operations to make use of a flexible integration approach while retaining the same level of complexity of the applications being integrated.

In order to enable effective monitoring and management of power supply networks according to the parameters collected in real time, electric power supply companies utilize various specialized systems, such as Supervisory Control and Data Acquisition System (SCADA), Distribution Management System (DMS), Automatic Meter Reading (AMR) and Geo-Information System (GIS) [4]. Each of the systems collects/measures from/on various power supply network elements and stores the data locally. Through EII mechanisms, implemented within any ESB solution, system-wide collected/measured data can be integrated to form a history of the power network structure, characteristics and behavior. Besides analyzing the previous system behavior, the data collected system-wide can be effectively used for the prediction of power supply network vulnerability, in terms of a prediction of defect probability for a particular network element. In addition to the electrical values gathered by the various systems, defect probability prediction requires collection of the value of any additional factors that can influence the functioning of power supply network elements. If additional factors are to be collected/measured, additional sensors should be installed into the existing infrastructure. Once the sensors are installed and integrated, the process of acquisition and analysis of the collected/measured values from additional sensors and existing systems should be delegated to a Sensor Web oriented system.

The Sensor Web represents a network comprised of sensor devices. Each sensor device has the ability to collect, process and display its measurements over the World Wide Web (WWW). Because of the ability to combine different types of sensors for the purpose of achieving different functionalities and producing different outcomes, potential Sensor Web usages are virtually limitless [5]. The vision of collecting real-time data from sensors distributed over a large sensing area, along with the ability to manipulate them over the World Wide Web, has initiated significant development and standardization of the Sensor Web concept. At the beginning of the current decade, standardization of the Sensor Web was started by the Open Geospatial Consortium (OGC), a leading organization in the field of developing open standards for geospatial and location services. OGC formed a working group that has developed a set of recommendations and specifications for proper development of a Sensor Web system. This set of documents, known as Sensor Web Enablement (SWE), comprises Web Service specifications and encodings [6]. As stated in the SWE specifications, Web services communicate with sensors, collect sensor measurements and poll them when necessary. SWE encodings are used to describe sensors' descriptions as well as models used for representing observations and measurements.

The Sensor Web has found various applications in different research and development fields, such as environmental monitoring and situational awareness. It has also been used in combination with various technologies. In many situations, Sensor Web has been successfully combined with Geo-Information Systems (GISs) that are capable of providing a visual dimension to the Sensor Web.

The purpose of this paper is to present a solution for power network vulnerability detection based on the Sensor Web and ESB integration architecture. The proposed solution is developed with the purpose of enabling prediction of power supply network vulnerability, in terms of a prediction of defect probability for a particular network element. The solution is based on GinisSense, an extended Sensor Web architecture [7,8], and it operates in an ESB environment combining Sensor Web and GIS technologies to enable prediction of electric power supply system vulnerability by determining defect probability for electric power supply network elements. This paper also presents an example of its usage and demonstrates our vulnerability prediction model on data collected from two different power supply companies.

## 2. Related Work

### 2.1. Sensor Web Solutions

The emergence of the Sensor Web concept dates from 1997 when Kevin Delin of NASA defined it as a system of wireless, intra-communicating, spatially distributed sensor pods that could be easily deployed to monitor and explore new environments [9,10]. Describing it as *a macro-instrument for coordinated sensing*, Delin pointed to its significance and potential for successful application in building environment observation-related systems [11]. Initially shaped at NASA's Jet Propulsion Laboratory for the purposes of the battlefield surveillance, the concept spread rapidly throughout diverse application areas. The essential idea of a Sensor Web-based system was to establish an intelligent sensor network that would enable its constituents to wirelessly communicate with each other, share data and other relevant information and act accordingly by sending collected information via Web to the end-user. Such behavior has proven to be very convenient and efficient for various purposes, which has resulted in a wide application range and a growing number of possibilities for exploiting Sensor Web-based systems.

The Open Geospatial Consortium (OGC), a leading organization in the field of developing open standards for geo-spatial and location-based services, has contributed significantly to the standardization of the Sensor Web concept. OGC has recognized early the potential of intelligent sensor networks and has formed a working group, named the Sensor Web Enablement working group [6], with the goal of developing a set of specifications and recommendations for introducing a Sensor Web system, named the Sensor Web Enablement (SWE) set of standards. The SWE initiative is focused on specifying standards for sensor discovery, gathering sensor observations, tasking sensors and eventing and alerting based on predefined rules and conditions. These functionalities are well defined through Web services and data model specifications, informally divided into two groups: information and interface models [12]. The information model specifies data models and encodings for describing sensors' capabilities, types of sensed data and events in observation process. The model contains the following XML based specifications: SensorML (SensorMarkup Language), Observation and Measurements (O&M) and Event Markup Language (EML). The first two are accepted as standards, while EML still has discussion paper status. SensorML defines a language for describing the capabilities of sensing devices: location, features that are being measured, measurement units, measurement intervals, manufacturer information and other details. Observation and measurements describes the formats of sensed data and gathered observations. SensorML and O&M together make up the standalone SWE Common 2.0 standard. EML represents a language for describing event patterns, based on Complex Event Processing rules, and it aims at incorporating rules-driven behavior into Sensor Webs.

The interface model of the SWE framework is comprised of different Web services' interface specifications that fully portray communication flows in a sensed environment: Sensor Observation Service (SOS), Sensor Event Service (SES), Sensor Planning Service (SPS), Sensor Observable Registry (SOR) and Sensor Instance Registry (SIR). SOS is the only service in the architecture that directly communicates with sensors in order to retrieve observations, as well as with client applications in order to present the gathered data. In that sense, SOS provides support for accessing sensed data. SPS, on the other hand, also communicates with sensors, but with the purpose of managing their behavior through tasking commands, thus enabling remote sensor control in a form of remotely changing sensor's settings regarding the measurement process. SOS and SPS together make up the SWE Service Model 2.0 standard. SES provides support for event-based alerting by enabling detection of defined complex patterns and thus prediction of events that in prior SWE versions could not be caught. SIR and SOR are in a discussion papers status, and are intended to provide support for the discovery features of Sensor Web systems. SIR is a catalogue service that provides functionalities for collecting, managing, transforming and transferring sensor metadata into conventional catalogues, making them available to users worldwide. SOR provides support for users in resolving observable phenomena identifiers through functionalities of retrieving available phenomena list, resolving the meaning of identifiers and finding related phenomena. The Discovery feature affects information models in addition to adding a Discovery profile to the SensorML standard that defines a minimum set of metadata elements and their structure that need to be provided in order for a sensor to be discoverable.

The integration of Sensor Web solutions into existing systems for the purposes of their enhancement has been a research topic for many groups and companies in the last few years. The best known are IrisNet [13], SenseWeb [14], GeoSWIFT [15], Vulcano Sensor Web [16], Abakus [17], SWAP Framework [18] and others. These systems are deployed in various domains but with the same purpose: to enable retrieval, processing and sensor data interpretation using geo-web interfaces.

- **IrisNet** is a network of smart sensors, divided into layers of sensing and organizing agents, and other monitoring devices that enable querying recent and historical sensed data. It aims at providing a general solution for building a World Wide Sensor Web and acts as a framework that could easily include new sensing device and their readings. IrisNet represents measured data in XML format and stores it in XML databases. The XPATH query language is used as a querying tool for retrieving information of interest either directly from the database or from available agents.
- **SenseWeb** is a Microsoft Research group's project whose main goal is providing a platform and a set of tools for quick and easy sensor data publishing [19]. The SenseWeb platform is comprised of a set of tools for data and metadata publishing, a spatial database for data indexing, an aggregator for data archiving and a GUI client that enables data filtering and reviewing of measurement results.
- **GeoSWIFT framework** is promoted as a distributed geospatial information infrastructure for the Sensor Web [20]. Built on a Web Services-based architecture it enables integration of different sensor types and data representation formats. The architecture involves a sensor layer, communication layer and an information layer. GeoSWIFT communicates with webcams as its sensing medium and processes measured values using a Sensing Server component.
- **The Volcano Sensor Web** project has been developed at NASA's Jet Propulsion Laboratory and is currently running with the aim of tracking the Earth's most active volcanoes [21]. The Volcano Sensor Web system is based on satellite images primarily received from Terra and Aqua satellites. Images taken by Moderate Resolution Imaging Spectrometer (MODIS) instruments are available nearly in real-time for regional coverage and 3–6 h from acquisition for global coverage. Combining them with *in-situ* sensors they have managed to develop an extremely sophisticated system for tracking volcano activities and reacting in case of possible danger.
- **Abacus** is a multi-agent system for managing radar data and providing decision support. It is built on a three-layered architecture, comprised of contribution, management and processing, and distribution layers. Each layer contains agents performing necessary operations for that particular layer: contribution layer's agents wrap physical sensors; management and processing layer's agents are responsible for processing data for a given spatial location or spatial sector; distribution layer's agents provide data visualization and broadcast warnings trough the web or via email [22]. Different decision rules could be defined through user interfaces and by using these rules it is possible to generate alarms for the processing agents.
- **The SWAP framework** is built on a multi-agent and ontological infrastructure. The multi-agent infrastructure provides inter-agent communication, while the ontological infrastructure provides the semantic wrapping necessary for the discovery, reuse and integration of Sensor Web data [16]. The SWAP architecture is designed to enable discovery and exploitation of sensor resources, sensor data fusion and context based information extraction. Each architecture layer (sensor layer, knowledge layer and application layer) has different agents responsible for successful collection and distribution of data to the subsequent level. The Sensor Layer is responsible for communicating with sensors, either directly or using intermediary services defined by the OGC Sensor Web Enablement specifications [6]. The knowledge layer has three types of agents that are responsible for capturing and storing expert knowledge. The application layer provides human and machine interfaces for interaction with the system.

### 2.2. Sensor Based Approaches in Power Supply Companies–Existing Solutions and Initiatives

Sensor Web systems relay on sensor networks comprised of orbital or terrestrial, fixed or mobile sensing units. *In-situ* sensor networks are most commonly used in Sensor Web concept implementation, as sensors are thus being placed directly in the sensing environment and are capable of providing immediate responses and performing measurements at any time. Geographical location plays an important role in sensors' descriptions as it provides the possibility of not only obtaining measured values of observed phenomena, but also of representing sensors' location on the map, thus providing a spatial extension to such observational systems. Due to the significant advances in sensor technology that have enabled development of smaller, cheaper and more efficient sensing devices, today's sensors could be placed anywhere in the observed environment, which has opened the door for involving Sensor Webs in many industrial systems. Power supply companies are among those which have found these types of systems to be very useful in monitoring and analysing a power network's state and especially in predicting its behaviour based on sensed environmental values. Specialized systems within company's Information System, such as SCADA, AMR and DMS generate large amounts of data that describe the power network's state and behaviour. Adding to them real-time and stored data received from field sensor networks, the amount of data becomes overwhelming and practically impossible for manual processing. There are only a limited number of initiatives attempting to resolve such issues.

Grilo *et al*. have explored the approach of using wireless sensor and actuator networks for improving electrical power grid dependability within the Wireless Sensor and Actuator Networks for Critical Infrastructure Protection (WSAN4CIP) project [23]. Placing sensors on the grid, particularly cameras equipped with infrared thermosensors, temperature sensors and light actuators, they have developed a system for improving the grid's safety and the dependability of the substations' components. Each time a camera detects a motion during a period when it is not expected to be any movements around the grid in the substation, it sends a video feed to the control centre, thus providing a mechanism for remote monitoring of human activity in secondary substations. At the same time, an infrared thermosensor sweeps the power transformation's critical elements, enabling the detection of a hotspot that would cause the alarm triggering, while the actuator turns on the lights in the substation. The temperature sensor measures the temperature of the substation's elements and provides a mechanism for remote monitoring of this feature's status. This sensor network was integrated with SCADA system, with the purpose of providing a unified interface to the human operators, and the entire system was deployed in a power distribution company in Portugal for the purposes of trial testing.

Another research project was focused on the utilization of wireless sensor networks for detecting mechanical failures in transmission lines [24]. This resulted from the fact that operators in the control centres only receive indication that an electrical fault has occurred without any further information regarding whether the fault is permanent or temporary as well as the type of the fault, estimated repair time, estimated repair effort *etc*. Such damage assessment was only possible by visual inspection, which is not always easy to carry out, especially when transmission lines are dispersed over large areas. Leon *et al*. have proposed a sensor based solution that could successfully address environment- (wind, snow, ice, flood, *etc*.) and human-related (accidents, terrorism) hazards. The solution involves placement and utilization of tension, displacement, acceleration and temperature sensors, installed in transmission line towers and capable of communicating wirelessly with control centres. Based on predefined values for each recognized hazardous situation, the system enables diagnosis of electrical faults, determining measures that could be automatically taken, acquisition of a complete physical and electrical picture and alerting once an extreme mechanical condition is identified on a transmission line.

The noticeable increase in frequency of blackouts during the last decade has influenced the research conveyed by the Mississippi State University that was related to wide area monitoring of an electrical system and its integration within the existing SensorNet management system [25]. SensorNet is a Sensor Web solution intended for real-time detection, identification and assessment of chemical, biological, explosive and other threats. Mohan *et al*. have proposed the solution that is based on OGC Sensor Web standards and Common Information Model (CIM) standard, as the one chosen for representation of electrical data. They have integrated CIM with OGC's O&M and SensorML representations, thus providing a common language model for describing different power supply systems' values and enabling fluent communication between heterogeneous data sources. The integration was performed between Sensor Web system and Phasor Measurement Units, which were installed at predefined positions of the electric power grid of Mississippi. The final outcome of the system was to provide information, rather than just data, describing the state of the power network's elements.

Based on the previously introduced initiatives in this area, it can be noticed that integration of electric power network with Sensor Web solutions could bring great benefits to power supply companies. The first two initiatives were concerned with utilization of sensor network regardless of communication standards. They are using their own communication models and solutions and are not relying on any known and publicly accepted recommendation or model specification. This significantly constrains the scalability of such solutions and as well imposes significant challenges towards their application in other companies. The third initiative is the closest to what we are trying to achieve: it relays on OGC SWE specifications for resolving issues related to communication with sensor network and uses CIM/XML communication model [26] for enabling integration between different systems of the power supply company. The system was tested in practise and proven to be very useful in resolving wide area monitoring challenges. However, the solution intended for addressing the needs of the ED Jugoistok power supply company in Serbia needs a wider approach considering the integration of company's subsystems. The communication is based not only on SCADA and sensor networks but also on GIS, DMS and AMR technical subsystems with sensor networks, which requires a more sophisticated integration approach. We have, therefore, expanded this research with integration-related challenges in power supply companies.

### 2.3. Vulnerability Prediction Models

Vulnerability prediction in power supply companies has been a subject of many research projects resulting with various different approaches that consider modeling of such predictions. Probably the most important aspect for analysis relates to cascading failures as series of failures that could lead to large-scale blackouts and other massive power grid damage. Each such failure is referred to as a contingency and the analysis of cascading contingencies is better known as N-x contingency analysis, where N represents a number of power grid components, while x is the number of simultaneous failures that occur on the observed network [27]. N-1 contingency analysis is rarely used in practice as is almost never the case that cascading failures are caused by a single credible contingency. Therefore, N-2 and higher cases are usually been questioned, as two or more simultaneous and seemingly individual events are more likely to cause cascading events. We will further present some common approaches to modeling vulnerability predictions in described environment.

Haidar *et al*. have been working on a vulnerability prediction model that uses vulnerability indices and relies on a neural network for pattern detection [28]. They have used vulnerability indices that are calculated based on power system loss (PSL) and possible loss of load (PLL). PSL index considers total system losses, generation losses due to generation outage, power line losses due to line outages, increases in total load and amounts of load disconnected and is conceptualized based on the fact that losses in a power transmission system are a function of both-system load and generation. The PLL index represents the possible loss of load due to the amount of load shed and is defined based on the idea that in an unpredictable situations, such as earthquake or flood, the operators would need to shed some load to ensure the safety of some core parts of the power system. The authors of this solution have used a probabilistic neural network approach for modeling vulnerability prediction of power network based on calculated values for previously explained vulnerability indices. Such an approach represents a fast intelligent solution capable of learning from experience and applying newly generated knowledge to the upcoming events.

In the attempt of finding ways for modernizing the U.S. electricity system, Rudin *et al*. have proposed a proactive plan based on a machine learning approach for New York City's (NYC's) power grid [29]. They emphasize the important shift from traditional *reactive* (fix when something goes wrong) to contemporary *proactive* (fix potential problems before they happen) modeling approach. The entire knowledge discovery process begins with the process of cleaning data coming from various sources (structured text, categorical data, numerical data, *etc*.) and integrating such cleansed data into a common database. Only then one or more machine learning algorithms could be applied on this integrated data, initiating the evaluation processes. The proposed model was applied, among others, on the ranking of the reliability of 1,000+ high voltage feeders in the NYC electrical system and determining the features that affect that reliability: overloads, power quality events such as voltage spikes, at-risk topologies and others.

Driven by the increased problem of power network instability, researchers at MIT have announced a new algorithm that efficiently identifies the most dangerous pairs of failures among the millions of possible failures in a power grid [30]. The algorithm was tested on a Polish power grid and the results have shown a promising speed of contingencies detection. The work on this algorithm is about to be published, and according to researchers that have worked on it, more extensive testing is needed in order to tune its performances even more.

In our research we have turned to a different approach, which is based on event modeling. We will present a prediction model that is based on sensor data fusion principles, events and rules for matching the patterns of events. Our model is flexible enough to easily adopt new rules for pattern matching in accordance with particular prediction needs. This means that each of the described approaches could effortlessly be exploited and built in into our model as core prediction implementation. In this sense, we could talk about our prediction model as a prediction framework for assessing the extent of power network vulnerability.

### 2.4. ESB-Based Information Integration on the Company Level

Although the need for enterprise application integration has been a popular topic for several decades now [2,31], true efforts towards resolving that issue have only now started to be seen with the increased appearance of data overloaded systems, such as power supply companies. The significant increase of business procedures and the amount of work processes in these companies over the last few decades, along with noticeable climate change and increased energy demands [32], have influenced a major growth of their Information Systems, seen through expansion of sub-systems, such as SCADA, AMR, GIS and DMS. Each of them generates large piles of data that are of importance for other sub-systems and are utilized for the purposes of integration with other system data. Taylor and Kazemzadeh emphasized the importance of SCADA, Outage Management System (OMS) and DMS integration, promoting improved operator efficiency, improved voltage management process, improved work coordination, reduced data maintenance efforts, improved operations, and integrated security analysis as most obvious integration benefits [4]. Given the fact that OMS is already integrated with GIS, Customer Information System, Work Management Systems and others, the proposed integration could considerably improve company's business procedures and increase its efficiency. The authors have also given an integration architecture that relies on a common network model, which enables data exchange between any two systems utilizing such a model.

Bernstein and Haas have provided a nice review of the integration tools and techniques, including data warehouse loading, virtual data integration, message mapping, object-relational mapping, document and portal management [31]. They are placing the need for standardized messages in the center of the integration challenge and are proposing XML as key standard in this area. However, when choosing an integration approach, many factors need to be considered: the size of the company, the number of integration participants, the amount of data flowing through the system on daily bases, the amount of communication with external partners, and many others [33]. Power supply companies are large companies responsible for managing energy usage for wide areas. That implies a large number of households that need to be served, a significant grid area, as well as a highly utilized Information System for internal business procedures. Adding to that the fact that company's information system is comprised of a number of sub-systems, particularly SCADA, GIS, AMR and DMS, as well as that each of them produces large amounts of data on a daily basis and could be considered as individual integration candidates, a standard point-to-point integration approach automatically must be left out, as it would require a large number of communication interfaces and eventually would lead to a complete communication deadlock [34]. A broker-based approach is also not reliable, as it implies one communication mediator that is responsible for managing the entire data traffic between all participants. In a system of such large dimensions, the broker component would very quickly become a bottleneck causing the crashing of an entire communication. Message oriented approaches are the most reliable ones in such cases as they are based on intelligent communication management where the integration component completely supervises the data exchange process, leaving the participant completely unaware of the entire process. Enterprise Service Bus (ESB) is the most known and most used massage-oriented integration technology that provides an intelligent bus for data flow and number of components responsible for packing/unpacking messages, determining the receiver address, validating message content, transferring message to its destination and implementing a plan for the entire communication process [3]. ESB is usually combined with SOA techniques, thus giving the best application results.

Such challenges were of interest for many big companies that have started to develop sophisticated solutions for bridging obstacles imposed by traditional heterogeneous system organization. IBM offers a palette of products for energy and utility companies, devoted to providing smarter solutions for transmission and distribution, customer and market operations, electricity generation, gas extraction or water resource management [35]. Recognizing the importance of integrating heterogeneous and distributed data sources of power supply companies, as well as the importance of introducing and supporting smart grids, IBM has developed a Solution Architecture for Energy and Utilities (SAFE), which incorporates SOA techniques with industry standards enabling companies to build flexible data integration solutions [36]. The solution is based on an Enterprise Service Bus approach and it incorporates two buses: standard enterprise bus and event processing or time-dependent event bus. The entire architecture is organized through three distinct and well-connected layers: local device layer, which is the lowest level in the processing hierarchy and is responsible for capturing and distributing new data, a time-dependent layer, whose responsibility is to perform complex event detection and thus to provide new insights, and an enterprise service layer, which is the highest layer responsible for business optimization and process integration. The described solution offers a broad range of benefits for utility companies: asset management, workforce management, information management, planning management, operations management, customer experience and revenue management. A number of users confirm the high quality of IBM's solutions. Uttarakhand Power Corporation Ltd. from India has turned to IBM in order to *seek a way to gain control over network and revenue management, with the aim of reducing service interruptions and outages, and better targeting energy theft* [37]. After applying the solution, the company has confirmed increased billing efficiency in terms of reduced time needed for performing operations, as well as better decision support for determining possible energy theft locations, while they were expecting to achieve under 20% technical and commercial losses in the following period. Austin Energy from Texas, USA, is another satisfied customer that incorporated IBM's solution seeking to improve service and reliability by changing the way of delivering electricity [38]. The company has gained a smart network which enabled gathering of new information, smart consumption monitoring, reducing energy usage and responding to outages more quickly and efficiently.

Microsoft has introduced a Smart Energy Reference Architecture in an attempt to offer to power supply companies a collaborative and integrated solution for smart grid management as a response to dynamic changes in these companies' business procedures [32]. The solution is based on an Enterprise Service Bus integration component and provides integration between internal and external enterprise applications, enterprise and network operation centers, enterprise and mobile users, users and devices, users and portals, portals and enterprise applications. Implementing entity aggregation, process integration and portal integration patterns, and relying on SOA techniques, they have developed a solution that gained trust with customers. Enspiria Solutions has embraced Microsoft's solution in order to improve business intelligence procedures. As the solution offers integration with ESRI GIS software, Enspiria Solutions have utilized *geospatially-oriented business intelligence about tree-caused outages to focus vegetation management exclusively on particular high outage areas*. AREVA is another customer that has benefited from this solution in improving visualization of integrated information. Particularly, AREVA has achieved integration of weather information with electricity grid on the geographical map, placing weather information as a new layer over the grid layer. Such visual integration has provided the company with significantly improved wide-area situational awareness capabilities for more efficient and reliable grid management.

Oracle has developed a set of software solutions with the aim to provide utilities with the end-to-end applications which should help utilities to get engaged in Smart Grid and Smart Metering initiatives [39]. Oracle Utilities Network Management System and Oracle Fusion Middleware offer utilities software functionalities that handle streamlining of business processes, alignment of business applications and visualization for embedded spatial capabilities. Also, solutions such as Oracle Utilities Customer Care and Billing and Oracle Utilities Meter Data Management, offer utilities a possibility to interact with their customers through experience initiatives [40]. For example, Lee County Electric Cooperative (LCEC) with 200,000 customers is trying to improve monthly bill delivery using Oracle Utilities Meter Data Management and Oracle Utilities Customer Care and Billing [41]. This not-for-profit electric distribution cooperative used Oracle Utilities Smart Grid Gateway to provide a single connection point between existing and future smart grid devices and applications.

All of the above mentioned solutions are without a doubt highly sophisticated and a result of years of research and development. They all are based on an ESB integration approach and SOA principles, and they all provide wide-area support for power supply companies. When developing a solution for ED Jugoistok, we have faced a concrete requests that among other have highly prioritized sensor data management and integration of technical subsystems with sensor networks. Therefore, it was of the highest importance to choose a model for sensor data inclusion as well as for sensor data fusion. As Sensor Web represents Web accessible sensor networks and is fully modeled and standardized, that was our obvious choice for successfully responding to such a formulated request.

## 3. The Need for Information Integration at the Jugoistok Power Supply Company

The ED Jugoistok Power Supply Company in Nis, Serbia, is responsible for power management of southeast Serbia and is organized through six sub-divisions, each covering a distinct area of the entire region. In 2012, within the project *Study on development feasibility of interoperable data exchange platform for the ED Jugoistok Information System*, we have performed analyses of the current state of the company's Information System through existing applications, their mode of usage and internal and external communications [42]. The applications of the company's Information System are divided in several logical groups: technical, business and Web portal. Technical systems are those responsible for managing network-related data: SCADA, AMR, DMS and GIS. They produce enormous amounts of data every day for different types of analysis and data processing. Business systems implement internal business procedures that are related to employees, documentation and other internal processes. Web portal represents an integration entry point and an external interface towards the entire system. It is built on a modular architecture where each module enables visualization of distinct features coming from the technical or business information systems.

**Figure 1.**
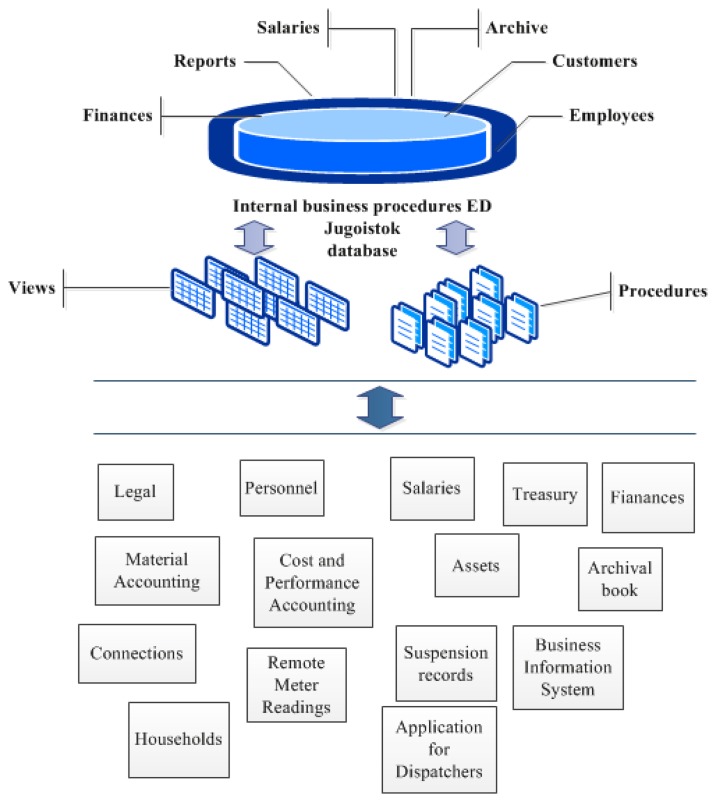
ED Jugoistok Information System: internal data exchange between applications that implement internal business procedures but are not directly connected.

The results of our internal communication analyses have demonstrated that in most cases applications are integrated via a database and directly communicate with each other. In rare cases the communication is performed “on paper”, that is, by hand entering data, or via a custom made communication service. This is the worst integration scenario as it requires maintenance of numerous communication interfaces between each application pair. Allowing applications to directly access to each other's database tables significantly endangers the safety of the company's Information System by exposing internal data as well as internal procedures. The entire analysis has pointed to four major situations that could be significantly improved by implementing integration based solutions:

1. *Internal data exchange between applications that implement internal business procedures and are not directly connected–*This is a typical scenario in the Information System of the Jugoistok power supply company for applications developed by distinct development companies. Those applications implement internal business procedures and communicate with each other via database views, stored procedures and other database-based integration techniques, as illustrated in Figure 1. There are many direct communication interfaces, and applications access directly to each other's data stored in a common database.
2. *Internal data exchange between applications that use separate databases*–This is a typical situation for applications that belong to technical subsystems: DMS, SCADA, AMR and GIS. Those applications function completely separately, each having its own database. Although there is a need for their communication, currently they do not communicate with each other at all.
3. *External data exchange–*This data exchange type that completely lacks in standardization. Having in mind that external communication is not of interest for this paper, we will not further elaborate on it.
4. *The need for a Web Portal–*A Web Portal represents an integration entry point for the company's Information System, providing the possibility of reviewing integrated information about the network by combining data from various technical subsystems. Based on a modular architecture, such a portal should provide a separate module for each technical subsystem with support for displaying integrated information: visualization module, network analysis, consumption per customer *etc*. Currently, there is no such portal for the Jugoistok power supply company. The company does have a web site, but its purpose is solely to provide an online company's presentation and a small subset of utilities for customers.

Based on the performed analysis and its results, the importance of developing an integration solution for the company was confirmed. Considering that a power network could consist of additional sensors, responsible for providing a wider observational context, the integration solution must include management of sensed data and provide a mechanism for fusing data coming from separate sensors with data obtained by technical subsystems. The architecture of the integration system should also include a Web Portal component, as an important integration enabler, pointing to its role and position in the communication process.

The next section of this paper presents the integration architecture for the ED Jugoistok power supply company, developed as a response to previously presented issues and challenges, and is followed by a use case, which demonstrates the application of such systems for predicting power network vulnerability.

## 4. System Architecture

Recent changes in the electric power supply domain have generated new requirements on the IT infrastructures in utility companies. The deployment of renewable energy sources (modern biomass, wind, solar, geothermal, and bio-fuels) have resulted in changes of the communication infrastructure and the development of even more IT systems which have to be integrated. Typically, electric utility companies relay on the following information systems to provide employees with a real-time comprehensive state of the distribution network [43]:

- Supervisory control and data acquisition (SCADA), Distribution Management System (DMS), Automatic meter reading (AMR)—these systems use traditional methods of data acquisition and control which rely on remote terminal units, power network analysers and remote metering infrastructure.
- Geo-information system (GIS)—this system is used for recording, maintenance and analysis of electric power supply network.
- Billing system (BS)—maintains data considering accounting, billing and connection of new households.
- Wide area monitoring and control system (WAMCS)—dynamic measurement systems based on the use of synchronized phasor measurement units (PMUs) [44].
- Wireless Sensor and Actuator Network (WSAN)—these network consist of appropriate sensors used to monitor key components within electric power supply network [23].

These systems have become indispensable in utility companies' daily business. Usually, each of the systems has a separate group of users whose requirements drive each of the systems towards becoming more complex and tightly coupled with various company business processes. This behavior results in different systems, duplicating the same data and functionalities, which in turn lead towards inconsistent data being used across the company. For example, since monitoring and management systems have the need for different power network analysis, it is necessary for them to have access to the technical data. Therefore, these systems store technical data locally, although such data exists in some of the technical systems (DMS, GIS). Also, DMS could be enhanced with the geographic component of the network elements for the purpose of easier and faster location of network hazards. Aside from geographical maps, this enhancement would impose an implementation of different geo-analysis, although these analyses already exist within GIS. Further, if it is necessary to monitor the status and quality of services, each of the network events should be coupled with customers that are affected by that event. In order to perform this type of coupling, data from DMS and one of the technical systems is necessary. Another common situation is the customer's request to increase the power of his/her household. Increased power usage introduces additional loads into the power network system so the critical power network components should be remotely monitored using appropriate sensors. The data collected from the sensors should be stored locally, usually within a system that belongs to a Sensor Web group. Additionally, collected data should be coupled with the geographical location and the component on which the measurements were made. At the same time, the data considering the location of different power network elements is stored separately within GIS while the components which are the subject of measurement are recorded as objects in SCADA, DMS or AMR systems.

**Figure 2.**
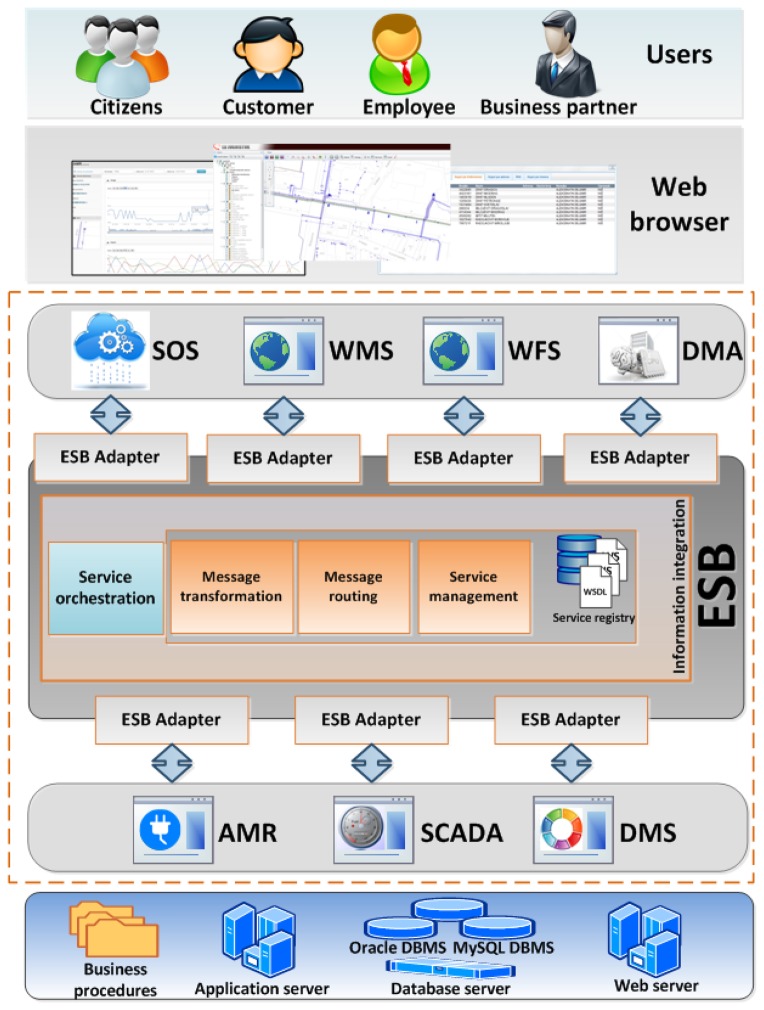
Service-oriented integration solution with ESB integration component.

In order to avoid data duplication and data inconsistency, there is a need to integrate existing information systems and applications, as well as new applications within and outside the utility company. A solution which meets this demand is the implementation of infrastructure for the information exchange that has to be flexible and extensible enough to meet future needs. This infrastructure has to provide a common model that can be used with various technologies and integration platforms. The analysis of integration patterns and contemporary integration technologies indicates that one of the best solutions in this field is the use of ESB integration components and Web Services as communication intermediaries [1,34]. A similar solution was used within the ED Jugoistok Niš electric power supply company. By analyzing the current state of the information system used in ED Jugoistok Niš, this utility company has decided to introduce a service-oriented integration solution with an ESB integration component, as shown in Figure 2.

The ESB component, which occupies the central part of the solution, enables the integration of applications, e.g., it enables data exchange between applications in a standardized and efficient way. The architecture presented herein consists of the following components:

- ESB Adapters—communication points responsible for accepting and forwarding incoming requests so that they can be processed; these components are also responsible for returning the processed data.
- Information integration—a component that implements all logics needed for processing the incoming requests. It consists of the following components:
  - Service orchestration—controls analysis of the received request and creates a plan for processing the request. This component implements communication rules and service mappings, which are used to generates the request execution plan. It also initiates the execution of the generated plan.
  - Message transformation—performs data validation and transforms data formats into the expected format.
  - Message routing—controls the message routing process. This component performs message routing, transforms communication protocols and implements two communication models: request/response and publish/subscribe.
  - Service management—implements operations used to register new services, modify and delete existing services. Therefore, the main responsibility of this component is to keep the Service registry component updated at all time.
  - Service registry—a registry of interface descriptions of all available services. The content of this directory is essential in creating a plan for the execution of user requests because it provides information considering all available services. Based on the service interface description provided by this component, the system can infer which services offer the required functionality.

As previously stated, this architecture supports two communication models:

- Request/Response model—solves the problem of the current data needs for a induvidual business process. This is the case with different applications within an information system which use the same database, but do not communicate directly. In this way, each application will be able to simply send a request to the bus and get the answer it needs without having the need for any information considering the method used to obtain the response.
- Publish/Subscribe model—one of the best solutions for the integration of data from the technical systems (AMR, DMS, GIS, SCADA). By implementing this model, different applications within the information system (for example a Web Portal) are given an opportunity to subscribe to different services and receive adequate information. Subscription types can be various:
  - Subscribe to receive data from a particular application.
  - Subscribe to receive data from a particular application according to a predefined criteria.
  - Subscribe to receive data considering a particular entity (for example current power network load) regardless of the application that generates the requested data (data can be obtained from a single or multiple applications).
  - Subscribe to receive data considering multiple entities according to predefined criteria (data can be data can be obtained from a single or multiple applications).

The communication flows either between different applications within the company's Information system, or between some of the technical systems and electric power network. In the first case, the communication is related to performing internal operations regarding the company's business procedures, while in the latter case, the communication is focused on collecting field data for the purposes of data processing, analysis and predictions by the technical systems and as such it represents the main focus of the research presented in this paper. Field data is made available via combination of transformer stations' measurement capabilities and additional sensors placed on carefully selected transformer stations' leads or any other parts of the network. Together they could be seen as a double sensor network comprised of a single sensors layer and transformer stations as intelligent sensors layer responsible for managing groups of interrelated single sensors. As such organized sensor network complies with the definition of a Sensor Web, we have incorporated the Sensor Web component Sensor Observation Service (SOS) that is essential for enabling communication with the sensor layer.

SOS is responsible for establishing direct communication with the sensor network in order to retrieve metadata and measurements from sensors and store them in a local database, as well as to provide this information to the end-user. To do so, SOS implements several operational profiles as recommended by the OGC SOS standard, among which Transactional, Result Handling and Enhanced ones are crucial for enabling successful communication. Sensors are calling SOS when they have new measurements or metadata and are placing their requests on ESB, following the previously described Request/Response communication model. The communication is XML-based, which means that all data coming from sensors is formatted according to well known, public XML schemas. After receiving a request, SOS performs appropriate operations and sends the response back via ESB. The request might be coming from sensors, when new data needs to be stored, or from a Web Portal, when the existing measurements and metadata are required by the end-user. In the latter case, depending on the request type, additional system components may be involved for the purposes of data analysis and pre-processing. That process is complex and is based on event-driven processing described through a set of pre-defined rules. The component that is in charge for implementing this part of communication is the Decision Making Agent (DMA).

### 4.1. GinisEDWeb— A Web-Based Solution for Visualization and Querying of Electric Power Supply Network Geospatial Information

GinisED Web is a part of the GinisED system—geographic information system for recording, maintenance and analysis of electric power supply network [45]. Being a Web GIS application, GinisED Web is an example of a Web 2.0 application used for visualization and querying of electric power supply network geodata. Thus, it can be classified into the group of GeoWeb 2.0 applications [46]. The architecture used for the development of GinisED Web application is modular. Due to its modular structure, GinisED Web can be easily expanded with additional functionalities. The creation of a modular WebGIS client with a rich user interface leads to the possibility of upgrading any developed solution. It also introduces a certain level of collaboration and the possibility of personalizing user-defined application interface parts.

**Figure 3.**
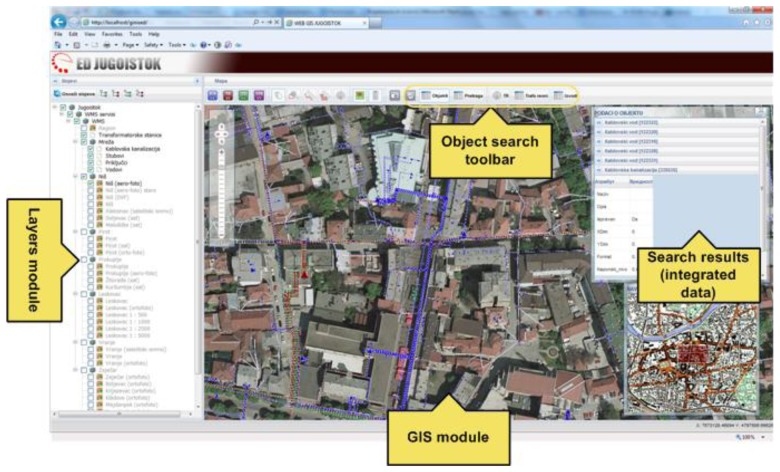
Web GIS application—client side modules' position.

GinisED Web has four basic modules: GIS module, layer selection module, objects search module (toolbar) and search (query) results module. The position of each module in the client is shown in Figure 3. The main module is a GIS module. This module implements a standard set of GIS functionality: increase and decrease scale of displayed map (default), scroll the map and positioning on the map, selection of part of map that needs to be shown, visualize entire map, reduced display of the complete map with marked part of map that is currently displayed in a certain scale. All other modules rely on this module and add new functionalities to the application, such as querying power supply network geo, joining thematic data from various sources, generating reports, *etc*.

Geospatial data and maps are visualized according to the selection performed within the layer selection module. Maps visualized by GinisED Web GIS application are divided in two groups of layers: the basic layers and layers of electric power supply networks elements. The basic layers represent different geographical maps which are obtained from the WMS via the ESB infrastructure. The obtained images are not transparent. They represent the foundation on which elements of the electric power supply network are displayed. Maps displaying elements of electric power supply networks are also obtained from WMS via the ESB infrastructure. They are transparent and can be combined with the maps on the client side according to the selections made within the layer selection module.

The GinisED Web application has a limited number of resolution levels that maps can be displayed in. This limits the number of images that can be requested from WMS. Because of the image number limitation, caching mechanisms can be implemented on both the client and server side. When a client requests an image from WMS via the ESB infrastructure, there is a high probability that the image has already been generated and cached because of another client's previous request. The number of previously requested, generated and cached images is limited by the required memory medium free space that WMS needs for image storage. Another possible solution is the usage of a Web Map Tile Service that already contains all the images that clients may possibly require. This solution completely eliminates the need for generating images on the server.

Among all electrical objects that GinisED Web GIS application displays, users in the Jugoistok power supply company are usually interested in one particular object or a group of objects. The significance that this object will be given, to a large extent depends on additional information attached to it. This information may change over time (dynamic) or may be unchanged during the prolonged period of time (static). Additional information is mostly from the non-geographical domain and is usually retrieved from a separate information sources via the ESB infrastructure. Characteristics of objects that are visualized on the map (in this case, the elements of electric power supply networks) vary depending on the object type. Object characteristics visualization is implemented using asynchronous requests to various information systems via the ESB infrastructure.

**Figure 4.**
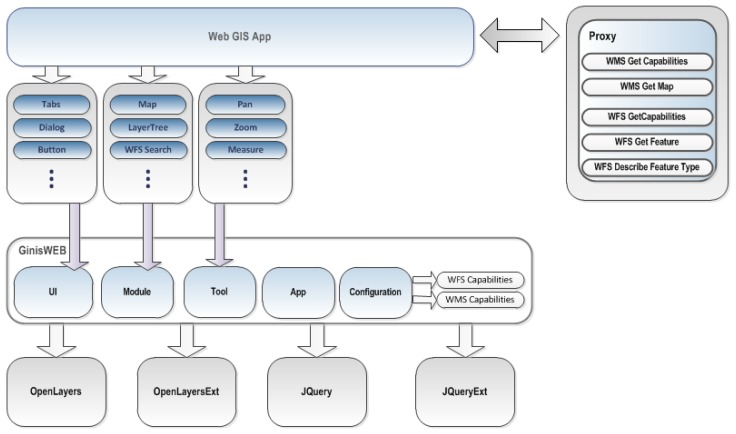
The architecture of the Web GIS client application.

It is important to emphasize that the GinisED Web application belongs to a group of medium thick clients which means that it combines advantages of a rich user interface with centralized data control. This is accomplished through the use of the GinisWeb framework, which relies on the modular architecture shown in Figure 4.

The GinisWeb is an AJAX-based framework which combines the best qualities from several Web AJAX GIS libraries into a single high level API framework. GinisWeb also adds custom functionalities considering electric power supply network data management on top of the integrated Web AJAX GIS libraries. The libraries integrated within the framework are:

○ OpenLayers—this is a framework for Web GIS application development and it is maintained by the Open Source Geospatial Foundation [47].
○ OpenLayersExt—is a collection of OpenLayers extensions which among other things adds support for the WMS 1.3.0 standard.
○ jQuery—is a framework primarily designed for Document Object Model (DOM) manipulation and it is compatible with a majority of contemporary Web browsers [48].
○ jQueryExt—is a collection of jQuery extensions.

The GinisWeb framework combines all these technologies into the following namespaces:

○ GinisWeb.UI–provides support for rich user interface creation by implementing various standard controls like dialogs, toolbars, panels, *etc*.
○ GinisWeb.Module–provides support for development of small modular units which are the main building blocks of a Web GIS application. The GinisWeb framework initially contains a set of modules which provide basic GIS functionalities. This module set among others includes the layer modules, map module, search module, *etc*.
○ GinisWeb.Tool–provides support for the creation of tools. The GinisWeb framework contains implementations of basic set of tools like panning, zooming, measuring, *etc*.
○ GinisWeb.App–provides support for creating Web GIS applications which unify modules and tools and enables their mutual interaction.
○ GinisWeb.Configuration–provides support for the creation of Web GIS configurations.

The GinisWeb framework is designed to enable simple linking and integrating with standard types of spatial Web services. Web GIS applications built using the GinisWeb framework communicate with external data sources (in this case the ESB infrastructure) through a Proxy service. The Proxy service is responsible for obtaining maps from custom providers and feature information from local data sources.

## 5. Vulnerability Prediction Model: Architecture and Implementation

DMA system component implements the decision-making logic based on sensed and electrical values of the power network's critical elements. In the proposed solution, DMA component implements the network hazard prediction logic. DMA is included as a service in the system architecture that performs entire communication with other system components via ESB, in accordance with described communication models. Usually, DMA is called by the Web Portal when the end-user needs prediction process to be initiated, but it could also be invoked by any other system component connected to the ESB.

**Figure 5.**
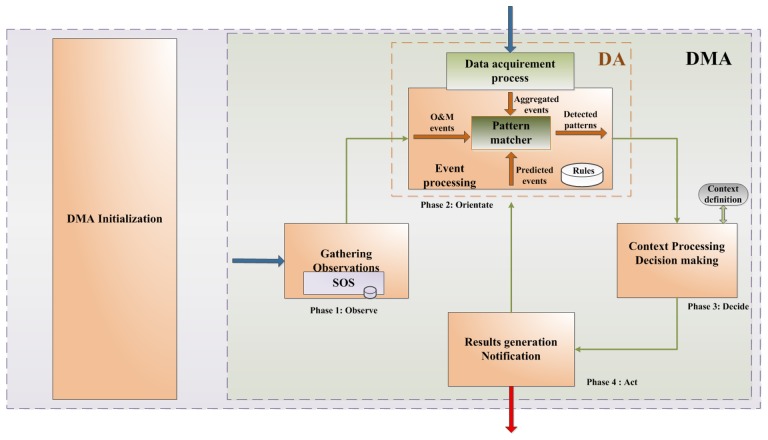
The architecture of the vulnerability prediction model based on sensor data fusion techniques.

Since the DMA is a rule-based system, the prediction logic is based on a predefined set of rules formalized to help identify potential hazardous events or situations that could lead to hazardous events. Predictions are generated by applying the rules on aggregated data. For this purpose, DMA component implements an adapted Omnibus data fusion model [49] and the architecture of the model is given at Figure 5. The fusion process, as described by the Omnibus model, is comprised of four separate phases: Observe, Orientate, Decide and Act. In the original model these phases make a cycle, where the final outcome from the Act phase is a new input into the Observe phase. For the purposes of its application in power supply companies and hazardous events prediction, we have broken the cycle and separated Act and Observe phases, and made a new connection from Act to Orientate phase. In such way we have enabled the events, which were detected as critical, to become new sources for the prediction process.

Prior to its utilization, DMA needs to be initialized. *The initialization* implies the following: (a) identifying transformer station leads that would be observed and obtaining their IDs; (b) obtaining IDs of customers connected to identified leads; (c) acquiring a history of electrical values for each identified customer and performing pre-calculations of those values.

*Data fusion* process starts with the *Observe phase* when the SOS is invoked to collect and store measurements from sensors placed on diverse electric network elements. Each observation represents one O&M event. The final result of this phase is represented with a set of O&M events matching pre-defined criteria, such as observational time period, geographic area, consumer, *etc*. Generated events are then sent to the next data fusion phase—Orientate.

The *Orientate* phase constitutes the core of the DMA component as it implements the event pattern recognition logic. The event prediction process is based on the analysis of sensed data, which consists of data collected by the SOS component (sensor measurements), and electrical values that represent the current state of the power supply network elements. The state of the power supply network elements in a particular moment (present or past) is represented as a set of electrical values gathered from the existing technical systems (AMM, SCADA, DMS). Data aggregation is performed as the process of coupling the state of each power supply network element with a single sensor or a sensor group that measures parameters which could affect the behaviour of the particular element. Data aggregation is delegated to the Data Aggregator (DA) component which is a part of the DMA component. In particular, within the Data Aggregator component, the data acquisition process can be observed as an independent process. Contrary to this, data aggregation process can be observed as an integral part of the DMA component. Therefore, data aggregation process represents a prerequisite for the prediction of the potentially hazardous events performed by DMA component.

DA is implemented as an event processing component that observes two event types: O&M events and aggregated events. O&M events are sensed events collected by SOS component and are representing particular observations. When a DA component is triggered, O&M events are retrieved from SOS's database, for the requested time period and in accordance with additional defined conditions. If necessary, a request might be sent from the SOS to retrieve the newest measurements from the sensor network. At the same time, DA starts communication with technical systems (AMR, SCADA, DMS) in order to obtain electrical values of the power supply network elements, matching only those elements that are of importance for the defined processing context. This communication is implemented by the Data acquisition process component whose responsibility is to generate aggregated events based on obtained electrical data. Once the events are ready, Pattern matcher starts matching them against predefined rules, stored in a Rules database, searching for patterns that would point to possible hazards in the power supply network. Figure 5 indicates the existence of a third source of events—predicted events. These are events that were predicted as critical in the previous DMA iteration and were sent to the Orientate phase from the Act phase. Their presence in this phase implies the necessity for another prediction process that would indicate potential vulnerabilities that could be caused by identified critical event.

Recognized patterns are sent to the *Decide* phase, where they are further matched against a set context. As a result, patterns that are considered to be critical are set as critical events, while those not considered critical in the defined context are ignored. The purpose of this phase is, therefore, to decide which are the critical patterns among all identified and to send them further into the next phase.

The *Act* phase initiates concrete actions. In the case nothing is detected it just sends a notification that there are no identified vulnerabilities. In case there is detected pattern it generates critical event and sends it to Orientate phase asking for additional prediction cycle in order to identify potential power network vulnerabilities caused by detected event. Once it receives this prediction from the Decide phase, it sends all detected events along with possible ones to those who required the analysis.

### 5.1. Vulnerability Prediction Model Implementation

The implementation of the prediction model was performed in two phases: the implementation of initialization activities and the implementation of pattern matching activities. The initialization activities are represented through the acquisition of a history of electrical values for each identified customer and pre-calculations of those values, while pattern matching activities are seen as rules applied on real-time gathered values. The variation of each of the observed values was determined in the form of a standard deviation. These deviations were calculated according to the following equations, where Equations (1) to (5) represent the initialization implementation, while the rest of the Equations (6) to (12) are part of pattern matching implementation:

μ_ea_—average value of individual customer's active energy (kW)
  (1) $$\mu_{\text{ea}}=\frac{1}{\text{N}}({\text{E}}_{\text{a}1}+{\text{E}}_{\text{a}2}+{\text{E}}_{\text{a}3}+\cdots +{\text{E}}_{\text{aN}})$$
μ_er_—average value of individual customer's reactive energy (kW)
  (2) $$\mu_{\text{er}}=\frac{1}{\text{N}}({\text{E}}_{\text{r}1}+{\text{E}}_{\text{r}2}+{\text{E}}_{\text{r}3}+\cdots +{\text{E}}_{\text{rN}})$$
μ_c_—average value of individual customer's consumption (kWh)
  (3) $$\mu_{\text{c}}=\frac{1}{\text{N}}({\text{C}}_{1}+{\text{C}}_{2}+{\text{C}}_{3}+\cdots +{\text{C}}_{\text{N}})$$
μ_mg_—average value of individual customer's maxigraph (kW)
  (4) $$\mu_{\text{mg}}=\frac{1}{\text{N}}({\text{MG}}_{1}+{\text{MG}}_{2}+{\text{MG}}_{3}+\cdots +{\text{MG}}_{\text{N}})$$
μ_t_—average temperature (°C)
  (5) $$\mu_{\text{t}}=\frac{1}{\text{N}}({\text{T}}_{1}+{\text{T}}_{2}+{\text{T}}_{3}+\cdots +{\text{T}}_{\text{N}})$$
F_σea_—Individual customer's active energy standard deviation (%)
  (6) $$F_{\sigma \text{ea}}=\sqrt{\frac{1}{N}\left[{\left(E_{a1}-\mu_{ea}\right)}^{2}+{\left(E_{a2}-\mu_{ea}\right)}^{2}+\cdots {\left(E_{aN}-\mu_{ea}\right)}^{2}\right]}$$
F_σer_—Individual customer's reactive energy standard deviation (%)
  (7) $$F_{\sigma \text{er}}=\sqrt{\frac{1}{N}\left[{\left(E_{r1}-\mu_{er}\right)}^{2}+{\left(E_{r2}-\mu_{er}\right)}^{2}+\cdots {\left(E_{rN}-\mu_{er}\right)}^{2}\right]}$$
F_σc_—Individual customer's consumption standard deviation (%)
  (8) $$F_{\sigma \text{c}}=\sqrt{\frac{1}{N}\left[{\left(C_{1}-\mu_{c}\right)}^{2}+{\left(C_{2}-\mu_{c}\right)}^{2}+\cdots {\left(C_{N}-\mu_{c}\right)}^{2}\right]}$$
F_σmg_—Individual customer's maxigraph standard deviation (%)
  (9) $$F_{\sigma \text{mg}}=\sqrt{\frac{1}{N}\left[{\left(MG_{1}-\mu_{c}\right)}^{2}+{\left(MG_{2}-\mu_{c}\right)}^{2}+\cdots {\left(MG_{N}-\mu_{c}\right)}^{2}\right]}$$
F_σt_—Temperature standard deviation (%)
  (10) $$F_{\sigma \text{t}}=\sqrt{\frac{1}{N}\left[{\left(T_{1}-\mu_{c}\right)}^{2}+{\left(T_{2}-\mu_{c}\right)}^{2}+\cdots {\left(T_{N}-\mu_{c}\right)}^{2}\right]}$$

The calculation of maximal allowed standard deviations for each of the observed values was a time consuming operation since it has to be performed for each of the customers that are being supplied with electric power from the observed transformer station. As defined by the prediction rule, transformer station lead is considered to be vulnerable if the average standard deviation of at least one of the electrical/ambient values in the observed period is greater than or equal to a given pre-calculated maximal allowed standard deviation. Once applied, this rule indicates whether a network element is vulnerable or not (Boolean (true/false) value):

(11) $$F_{\text{predictedperlead}}=\frac{\sum_{i=0}^{k}F_{\sigma ea}}{k}\geq F_{q\sigma ea}\cup \frac{\sum_{i=0}^{k}F_{\sigma er}}{k}\geq F_{q\sigma er}\cup \frac{\sum_{i=0}^{k}F_{\sigma c}}{k}\geq F_{q\sigma c}\cup \frac{\sum_{i=0}^{k}F_{\sigma mg}}{k}\geq F_{\text{qmg}}\cup \frac{\sum_{i=0}^{k}F_{\sigma t}}{k}\geq F_{qt}$$

According to the data fusion model, the process of determining the vulnerable power network leads is performed through the following stages:

1. Identify transformer station leads which will be observed. DMA components send a request to WFS via ESB and receives a set of lead IDs within WFS response (*e.g*., within a GML document which contains the description of transformer station leads including lead IDs). For each of the identified leads, DMA component acquires a set of IDs of customers connected to transformer station lead by invoking WFS via ESB. For each of the customers, DMA component acquires a history of electrical values via ESB from SCADA and AMR systems. Electrical values are gathered for a one-month period and used to calculate average electrical values for each of the observed values, according to Equations (1) to (5).
2. Observe—This phase collects sensor observations for individual transformer station leads. DMA component invokes SOS component via ESB to acquire temperature readouts. Once received, temperature readouts (received in the form of O&M events) are stored localy, within SOS component's database, and used for the vulnerability prediction in the following phases.
3. Orientate.
  - Acquire O&M events from the SOS component's local database (according to predefined parameters, *e.g*., for each of the observed transformer station leads).
  - Acquire electrical values for each of the identified customers. Electrical values are gathered by invoking AMR and SCADA components via ESB.
  - Couple temperature readout for a single transformer station lead with each customer connected to the observed lead.
  - If any predicted event exists (possible output from Act phase), include predicted events into the vulnerability calculation.
  - Detect event patterns according to a set of predefined rules stored within the Rules database. In this use case, a set of predefined rules used for event pattern detection is represented by Equations (6) to (10). Event patterns are calculated for each customer according to Equations (6) to (10). In case of the existance of the predetected events, received from the Act phase, which corresponds to an event pattern, in that case the event pattern calculation is skipped and considered to be calculated.
  - For each of the customers perform phases 4 and 5.
4. Decide—Match detected event patterns with the current context. In this use case, the context is used to determine lead vulnerability and is represented by Equation (11).
5. Act—Detected critical event are forwarded to the next round of calculations within the Orientate phase in order to determine the pottential consequences of the detected events. As the output, Act phase matches all critical events to transformer station leads that these events were detected for. The response is in the form of a set of transformer station lead IDs encoded within a GML document. Generated GML document is sent to the requesting component via ESB.

## 6. Feasibility Testing

In order to test the feasibility of the presented architecture and sensor data fusion model, we have performed two feasibility testing phases: verification of a value distribution model of the electric values used for the prediction of power supply network vulnerability and a functional testing of the proposed solution in laboratory conditions.

### 6.1. Feasibility Testing Phase 1: Verification of a Value Distribution Model of the Electric Values

During the first feasibility testing phase, for the purpose of verifying a value distribution model of the electric values, genuine power load data was gathered from two electric power supply companies: ED Jugoistok Niš (Serbia's local electricity transmission system operator) and Elia Bruxelles (Belgium's electricity transmission system operator). We were granted access to the AMR system belonging to ED Jugoistok Niš, so the data belonging to ED Jugoistok Niš was acquired directly from AMR system. As for the data belonging to Elia Bruxelles, this data is publicly available and was downloaded from the company's official Web site (download page: http://www.elia.be/en/grid-data/data-download).

In both cases, verification of the value distribution model was performed using electric power load data reported on 1 January 2013. The format of the data provided by these two companies differs in the way different companies represent total electric power load. Also, data granularity level differs for the two companies. For example, the sampling period of the data acquired from ED Jugoistok Niš was 1 h, which resulted in a small number of values that can be used for the model verification. To create a more appropriate testing data set, data reported on 1 January 2013 by ED Jugoistok Niš was extended with 14 successive readouts from the next day (until 2:05 PM on 2 January 2013). The resulting testing data set is shown in Table 1. As for the Elia company, the sampling period was set to 15 min which formed an appropriate testing data set, shown in Table 2.

Since the range of the values used within the two testing data sets differs, it was necessary to perform ordering and normalization of deviations between each of the testing data sets values and the mean value of power load for each of the testing data sets. Values calculated in this manner are designated as normalized power load values, and they are displayed in the last column of Table 1 and Table 2. Therefore, these columns represent normalized power load value distribution.

**Figure 6.**
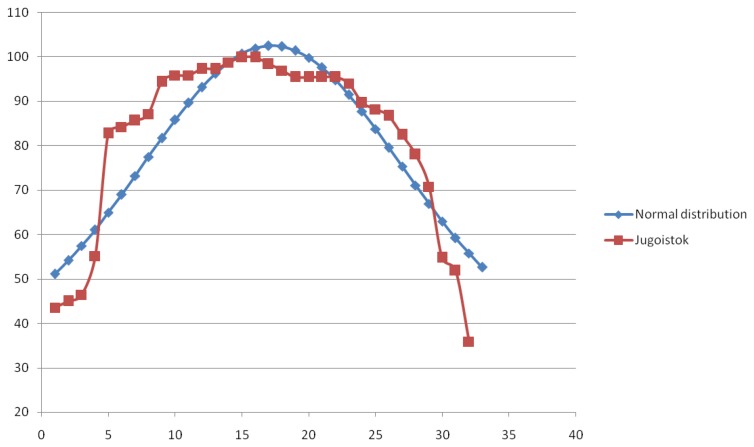
Normalized power load value distribution compared to the normal (or Gaussian) distribution for the given range of values—source: ED Jugoistok Niš.

**Figure 7.**
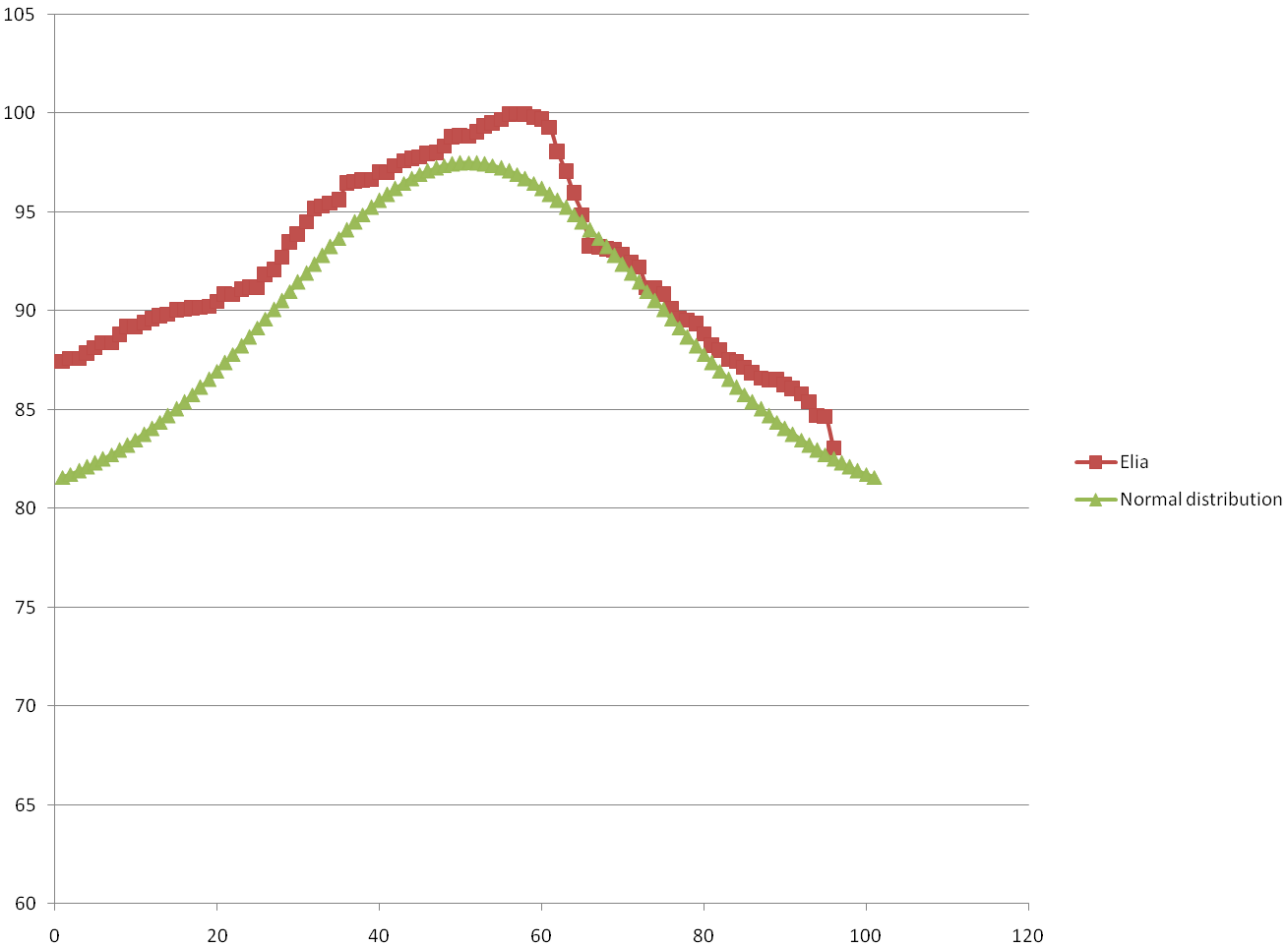
Normalized power load value distribution compared to the normal (or Gaussian) distribution for the given range of value—source: Elia Bruxelles, Belgium.

Since the vulnerability prediction model uses standard deviation (which assumes a normal (Gaussian) distribution of the observed values) to predict a defect probability for a particular network element, normalized power load value distribution for each of the companies was compared with an ideal Gaussian distribution of the observed values for the given range of values. The results of this analysis are displayed in Figure 6 for the ED Jugoistok Niš company, while Figure 7 represents the analysis results for the Elia company.

It is noticeable that normalized power load value distribution deviates from the normal (or Gaussian) distribution. These deviations may occur due to different factors: the status of individual power system elements, a relatively small sample of values used in the verification, a large sampling time or habits of consumers in a particular part of the power system. Nevertheless, the comparison shows that the normalized power load value distribution follows the Gaussian distribution for the observed cases and indicates that the standard deviation can be used as an indicator of vulnerability of individual power network elements.

### 6.2. Feasibility Testing Phase 2: Functional Testing of the Proposed Solution

In the second feasibility testing phase, we have prepared an environment simulating the working conditions in the ED Jugoistok Niš electric power supply company. In attempt to make the simulation environment as similar as possible to real conditions, we were assisted by external experts from the ED Jugoistok company. External experts in the field of electricity distribution participated in the creation of rules used for the prediction of power supply network vulnerability. Their assistance was very significant for the determination of values that can be considered critical for any of electrical and ambient values which were monitored during the simulation.

We have simulated a variation of electrical and ambient values on the leads for the predefined transformer station and performed the vulnerability prediction on the basis of one defined rule. The simulation included the monitoring of the following electric and ambient values for the predefined transformer station: active energy of individual customers connected to the transformer station leads (E_A_ – kW), active energy of individual customers connected to the transformer station leads (E_R_ – kW), consumption of individual customers connected to the transformer station leads (C – kWh), maxigraph of individual customers connected to the transformer station leads (MG – kW) and the temperature measured in the vicinity of individual transformer station leads (T – °C). The monitoring was performed for the transformer station “CRVENI KRST” 35 kV/10 kV, which supplies electric power to CG & GIS Lab facilities. In the ED Jugoistok company, SCADA and AMR systems are responsible for collecting electrical values. Collected data is stored locally within each of the systems and can be from other (IT) systems through ESB infrastructure. For simulation purposes, the necessary subset of collected data along with communication infrastructure was transferred to the laboratory conditions, so the electrical values were collected from the local data sources. As suggested by experts from ED Jugoistok, the time between two measurements (sample rate) for SCADA and AMR systems was set to 15 seconds. The same sample rate was used to collect temperature readings e.g., the same period of time was necessary to elapse between two invocations of SOS component used for collecting sensor measurements.

As suggested by external experts, February was chosen for this calculation because the electrical and ambient variations are expected to be largest during this period in one year. Standard deviations calculated for the month of February were then used as maximal allowed variations for each of the observed values. The average value of the standard deviation was calculated during the sampling period which was limited to 30 min. Once these values were determined, simulation was started and vulnerability prediction rules could be applied.

The vulnerability prediction rule, Equation (11), was defined for a single power network lead connected to the observed transformer station, in this case the transformer station “CRVENI KRST” 35 kV/10 kV. Customers were divided according to power network leads they are connected to. Temperature readouts were carried out for individual power network leads and were used within the prediction rule in combination with the values determined for individual customers connected to particular power network lead.

After vulnerable network leads are identified, they will be visualized through Web GIS application and marked red, while the rest of the leads will be marked green. This process is performed through the following stages:

1. Web GIS application acquires a set of identifiers of vulnerable network leads from the DMA component via ESB.
2. Web GIS application sends *GetFeature* request to WFS component via ESB. This request contains a set of all lead IDs.
3. WFS component generates a GML document which contains a description of all vulnerable leads including a geometry for each of the leads. WFS component sends the GML document to the Web GIS application via ESB.
4. Web GIS application receives the GML document and extracts geo-objects which represent all power network leads. Web GIS application creates a new layer which consists of the extracted geo-objects.
5. On the basis of the IDs of the obtained geo-objects, Web GIS application detects all vulnerable power network leads.
6. Web GIS application visualizes all power network leads. Vulnerable network leads are emphasize by being marked red while the rest of the power network leads are being marked green.

Web GIS application used during simulation proposes is depicted in Figure 6. This application represents a modification of the Web GIS application which is currently being used in the ED Jugoistok Niš company and it was also developed in the CG&GIS Lab.

**Figure 8.**
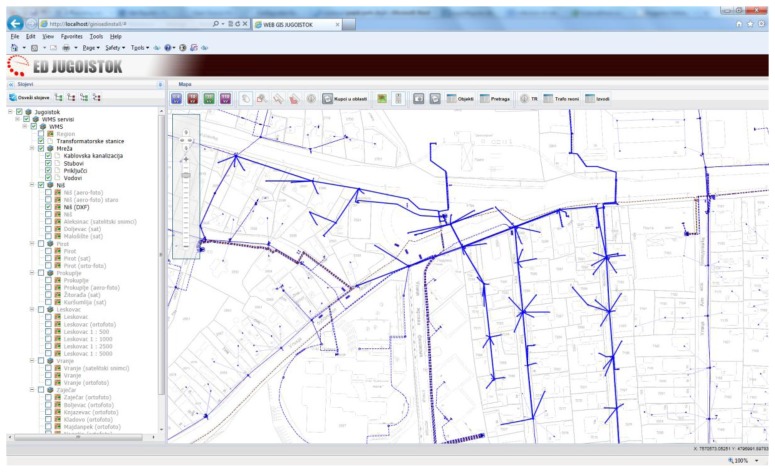
A part of the electric power supply network in the city of Niš which was under observation during the simulation process.

As shown in Figure 8, Web GIS application visualizes electric power supply network in the city of Niš and emphasizes the part of the network which was under observation during the simulation. Power network elements which were observed (transformer station leads) were obtained from WFS component in the form of GML document. On the basis of this document, a separate vector layer was created, marked blue and displayed on top of raster layer that visualizes the whole electric power supply network in the city of Niš. As previously stated, observed power network leads are connected to the “CRVENI KRST” 35 kV/10 kV transformer station.

**Figure 9.**
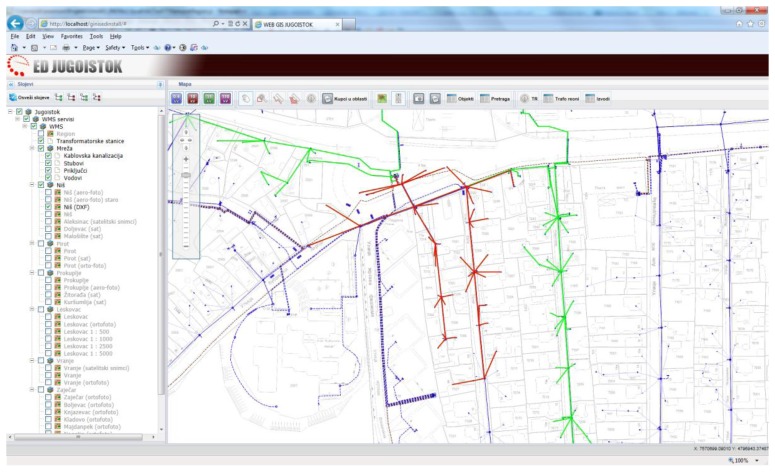
Simulation results—potentially vulnerable power network leads identified for the observed part of the electric power of the city of Niš.

After the DMA component of the adapted GinisSense system had analyzed sensor measurements and electrical values obtained from the SOS, AMR and SCADA components, potentially vulnerable power network leads were determined and marked red. Power network leads that were not determined to be vulnerable were marked green. The result is shown in Figure 9. As it can be observed, approximately half of the power network leads are considered vulnerable. This result is a consequence of the relatively short period of observation, as well as low values that were set for the maximal allowed standard deviation at the beginning of the test (1% variation for all standard variations).

## 7. Conclusions

The purpose of this research was to enable the prediction of electric power supply system vulnerability by determining defect probability for electric power supply network elements. As a result, we have developed a specialized system based on the components of GinisSense architecture. Implemented system operates in an ESB environment and enables the coupling of data gathered from GIS, specialized IT systems and sensors, that will be processed within an intelligent rule-based component. Data processing results are displayed through a Web GIS application which emphasizes potential power network element vulnerability by applying appropriate display style upon the power network elements. Since the vulnerability prediction is rule-based, the system's function can be altered to correspond to different users' needs and companies' requirements by changing the rules used in the prediction process. Once it is adjusted to current requirements, the described system could significantly enhances the quality of service by predicting the potentially hazardous events within the power supply network and offer employees the possibility to perform timely planned actions.

Simulation of the power supply system operating conditions in the laboratory demonstrated the feasibility of our architecture and sensor data fusion model. Although simulation results prove that vulnerability of power network elements can be predicted, at the same time, this simulation opened up a number of opportunities for future research and development. Our future work should primarily focus on developing extensions of prediction logic, as well as improving data fusion process. The system should be enhanced by allowing users to have more control over the prediction rules, e.g., by giving users the ability to change sampling rates and define maximal values for each of the standard deviations. These changes should be made for the purpose of improving flexibility of the system.

Currently, the presented system has the ability to perform a vulnerability prediction for individual leads of the selected transformer station. Further development will enable a vulnerability prediction for all leads belonging to an arbitrary geographical area. We plan to develop this functionality by implementing a tool that will allow users to define a geographical area which will be taken under observation. By taking advantage of this tool, users will be able to select a geographic area that includes different power supply network leads which are connected to various transformer stations. Also, we plan to focus on defining additional prediction rules, including spatio-temporal rules and rules that will be based on the use of previously determined vulnerability of individual network elements. Our aim is to store calculated vulnerability of network elements and use it to create vulnerability history for different power supply network elements. By creating permanent vulnerability history, the system could be given an opportunity to use these values as an additional input in both data fusion and vulnerability prediction processes.

## Figures and Tables

**Table 1.** Verification of electric values distribution model—source: ED Jugoistok Niš.

**Sampling Time**	**Power Load Increase—E (kW) (1 h Samples, 01/01/2013)**	**(E-μ)^2^**	**Power Load Increase—E (kW) (1 h Samples Ordered)**	**Normalized Power Load Value Distribution NORM (E/μ) (%)**
7:05	0.31	0.143451563	0.3	43.56
8:05	0.3	0.151126563	0.31	45.01
9:05	0.57	0.014101563	0.32	46.46
10:05	0.6	0.007876563	0.38	55.17
11:05	0.69	1.5625E-06	0.57	82.76
12:05	0.66	0.000826563	0.58	84.21
13:05	0.67	0.000351563	0.59	85.66
14:05	0.72	0.000976562	0.6	87.11
15:05	0.71	0.000451562	0.65	94.37
16:05	0.72	0.000976562	0.66	95.83
17:05	0.73	0.001701562	0.66	95.83
18:05	0.77	0.006601562	0.67	97.28
19:05	0.81	0.014701563	0.67	97.28
20:05	0.78	0.008326562	0.68	98.73
21:05	0.72	0.000976562	0.69	99.82
22:05	0.89	0.040501563	0.69	99.82
23:05	0.32	0.135976563	0.7	98.37
0:05	0.58	0.011826563	0.71	96.91
1:05	0.38	0.095326563	0.72	95.46
2:05	0.59	0.009751563	0.72	95.46
3:05	0.65	0.001501563	0.72	95.46
4:05	0.66	0.000826563	0.72	95.46
5:05	0.68	7.65625E-05	0.73	94.01
6:05	0.76	0.005076562	0.76	89.66
7:05	0.84	0.022876563	0.77	88.2
8:05	1.13	0.194701563	0.78	86.75
9:05	1.02	0.109726563	0.81	82.4
10:05	0.7	0.000126562	0.84	78.04
11:05	0.69	1.5625E-06	0.89	70.78
12:05	1	0.096876562	1	54.81
13:05	0.72	0.000976562	1.02	51.91
14:05	0.67	0.000351563	1.13	35.93
	μ—average value of power load increase		0.68875	
	Individual customer's consumption standard deviation		0.183622405	
	Individual customer's consumption standard deviation (%)		26.66	

**Table 2.** Verification of electric values distribution model—source: Elia Bruxelles, Belgium.

**Sampling Time**	**Power Load—E (kW) (15 min Samples, 01/01/2013)**	**(E-μ)^2^**	**Power Load—E (kW) (15 min Samples ordered)**	**Normalized Power Load Value Distribution NORM (E/μ) (%)**
0:15	8386188	6.27434E + 11	6641657	87.46
0:30	8269031	4.55558E + 11	6652119	87.6
0:45	8118750	2.75278E + 11	6652653	87.6
1:00	7983364	1.51542E + 11	6668676	87.81
1:15	7904270	96217428976	6693801	88.14
1:30	7744050	22490804065	6712388	88.39
1:45	7648916	3006914924	6712443	88.39
2:00	7503948	8123895723	6740660	88.76
2:15	7426564	28061830121	6772199	89.18
2:30	7332042	68664257369	6772408	89.18
2:45	7223951	1.36996E + 11	6786130	89.36
3:00	7126662	2.1848E + 11	6801238	89.56
3:15	7042309	3.04452E + 11	6813318	89.72
3:30	6970847	3.8842E + 11	6821739	89.83
3:45	6922830	4.50577E + 11	6835372	90.01
4:00	6871110	5.22687E + 11	6841591	90.09
4:15	6843553	5.63292E + 11	6843553	90.12
4:30	6801238	6.28599E + 11	6848978	90.19
4:45	6786130	6.52784E + 11	6851869	90.23
5:00	6772199	6.75489E + 11	6871110	90.48
5:15	6813318	6.0959E + 11	6897147	90.82
5:30	6821739	5.96512E + 11	6898156	90.84
5:45	6841591	5.66241E + 11	6915838	91.07
6:00	6848978	5.55178E + 11	6922141	91.15
6:15	6898156	4.84311E + 11	6922830	91.16
6:30	6897147	4.85717E + 11	6970847	91.79
6:45	6915838	4.60013E + 11	6989324	92.04
7:00	6851869	5.50878E + 11	7042309	92.73
7:15	6693801	8.10503E + 11	7096969	93.45
7:30	6641657	9.07111E + 11	7126662	93.84
7:45	6652119	8.87292E + 11	7178054	94.52
8:00	6652653	8.86286E + 11	7223951	95.13
8:15	6668676	8.56374E + 11	7238719	95.32
8:30	6712443	7.77285E + 11	7249223	95.46
8:45	6740660	7.28327E + 11	7258498	95.58
9:00	6712388	7.77382E + 11	7323823	96.44
9:15	6772408	6.75146E + 11	7332042	96.55
9:30	6835372	5.75639E + 11	7334398	96.58
9:45	6922141	4.51503E + 11	7340466	96.66
10:00	6989324	3.65731E + 11	7365044	96.98
10:15	7096969	2.4712E + 11	7366000	97
10:30	7178054	1.73078E + 11	7393162	97.35
10:45	7258498	1.12616E + 11	7411918	97.6
11:00	7334398	67435081957	7416310	97.66
11:15	7393162	40368306429	7426564	97.79
11:30	7436062	24969895723	7436062	97.92
11:45	7520541	5408081041	7438990	97.96
12:00	7541671	2746772068	7469019	98.35
12:15	7592592	2216097.431	7500637	98.77
12:30	7570399	560820842.7	7503948	98.81
12:45	7601706	58145867.31	7506751	98.85
13:00	7611260	295129851.7	7520541	99.03
13:15	7586922	51246359.31	7541671	99.31
13:30	7506751	7626468861	7553274	99.46
13:45	7469019	15640417864	7570399	99.69
14:00	7411918	33183233332	7586922	99.91
14:15	7366000	52020785755	7592592	99.98
14:30	7340466	64320393865	7601706	99.9
14:45	7249223	1.18927E + 11	7611260	99.77
15:00	7238719	1.26282E + 11	7619268	99.67
15:15	7323823	73039200762	7648916	99.28
15:30	7365044	52457789906	7744050	98.03
15:45	7416310	31602406224	7819157	97.04
16:00	7438990	24053111656	7904270	95.92
16:15	7500637	8731716893	7983364	94.87
16:30	7553274	1665183194	8105570	93.26
16:45	7619268	634402285.2	8110100	93.2
17:00	7819157	50659360516	8118750	93.09
17:15	8121235	2.77892E + 11	8121235	93.06
17:30	8348979	5.69872E + 11	8135626	92.87
17:45	8489386	8.01572E + 11	8171951	92.39
18:00	8570026	9.52469E + 11	8184307	92.23
18:15	8621027	1.05462E + 12	8264256	91.18
18:30	8638739	1.09131E + 12	8269031	91.11
18:45	8653844	1.1231E + 12	8292219	90.81
19:00	8610851	1.03382E + 12	8348979	90.06
19:15	8594097	1.00003E + 12	8386188	89.57
19:30	8539776	8.9434E + 11	8389422	89.53
19:45	8546493	9.07089E + 11	8405874	89.31
20:00	8507138	8.33674E + 11	8441642	88.84
20:15	8405874	6.59008E + 11	8489386	88.21
20:30	8292219	4.87397E + 11	8507138	87.98
20:45	8264256	4.49135E + 11	8539776	87.55
21:00	8184307	3.48367E + 11	8546493	87.46
21:15	8171951	3.33934E + 11	8570026	87.15
21:30	8135626	2.93271E + 11	8594097	86.83
21:45	8110100	2.66276E + 11	8610851	86.61
22:00	8105570	2.61621E + 11	8621027	86.48
22:15	8389422	6.32568E + 11	8621582	86.47
22:30	8672769	1.16357E + 12	8638739	86.24
22:45	8758013	1.35474E + 12	8653844	86.04
23:00	8701556	1.2265E + 12	8672769	85.8
23:15	8879615	1.6526E + 12	8701556	85.42
23:30	8754587	1.34677E + 12	8754587	84.72
23:45	8621582	1.05576E + 12	8758013	84.67
24:00	8441642	7.1836E + 11	8879615	83.07
	μ—average value of power load		7594080.656	
	Individual customer's consumption standard deviation		675981.8861	
	Individual customer's consumption standard deviation (%)		8.90143148	
